# Heat Beyond the Kitchen: Embodied Strain, Time Poverty, and the Life-Embedded Nature of Employee Experience in Culinary Labour

**DOI:** 10.3390/bs16060905

**Published:** 2026-06-03

**Authors:** Derya Alimanoğlu Yemişçi, Zehra Dilistan Shipman, Nasrettin İlhan, Ümit Deniz İlhan

**Affiliations:** 1 Department of Labor Economics and Industrial Relations, Manisa Celal Bayar University, Manisa 45140, Türkiye; derya.yemisci@cbu.edu.tr; 2 Department of Gastronomy and Culinary Arts, İstanbul Nişantaşı University, Istanbul 34398, Türkiye; dilistan.shipman@nisantasi.edu.tr (Z.D.S.); nasrettin.ilhan@nisantasi.edu.tr (N.İ.); 3 Department of Business Administration, İstanbul Nişantaşı University, Istanbul 34398, Türkiye

**Keywords:** employee experience (EX), embodied labour, time poverty, social reproduction, work–life interface, occupational well-being, culinary labour

## Abstract

This study examines how employee experience (EX) in professional kitchens is constituted through the interplay of embodied strain, temporal constraints, care responsibilities, and the meanings attached to work. Drawing on an embedded single-case study conducted in the main kitchen of a five-star city hotel in Istanbul, the study is based on in-depth interviews with workers across hierarchical levels. The findings show that physical strain, long and irregular working hours, and non-work care burdens are not experienced as separate challenges, but as mutually reinforcing pressures that extend into everyday life. Based on these findings, the study reconceptualizes EX as a life-embedded psychosocial process constituted across bodily, temporal, and extra-organizational domains. Within this framework, the study introduces two conceptual mechanisms: embodied time poverty, which captures how time pressure becomes intertwined with bodily exhaustion and limited recovery, and the hidden social burden of culinary labour, which explains how care responsibilities and everyday life demands become constitutive of EX. The findings further show that the discourse of passion operates as a meaning-making and normalizing mechanism that legitimizes demanding working conditions while obscuring their cumulative psychosocial costs. Overall, the study contributes to the EX literature by offering a more integrated understanding of work, well-being, and everyday life in labour-intensive settings.

## 1. Introduction

Professional kitchens have increasingly been represented—both in academic and popular discourse—through narratives of creativity, craftsmanship, passion, and professional dedication (Angelini et al., 2025; Kuhn et al., 2025). Within this framing, culinary work is often portrayed not merely as technical production, but as an identity-defining practice associated with mastery, sacrifice, and commitment (dos Anjos et al., 2026; Vu et al., 2023). While these representations contribute to the cultural valorization of kitchen labour, they also risk obscuring the material, organizational, and psychosocial conditions through which such labour is sustained (Seyitoğlu et al., 2023). In practice, professional kitchen work is characterized by intense physical demands, long and irregular working hours, operational pressure, and limited opportunities for recovery (Cerasa et al., 2020; Melaku et al., 2024). These conditions frequently extend beyond the workplace through fatigue, time scarcity, reduced social participation, and disrupted everyday life (Geyser et al., 2023; Saito et al., 2025). Accordingly, the experience of working in professional kitchens cannot be understood solely in relation to tasks, performance expectations, or organizational responsibilities, but must also be examined through the embodied, temporal, and psychosocial conditions under which work is lived.

The literature on embodied labour provides an important analytical lens for understanding this condition. Research in organizational studies, sociology of work, and service labour increasingly emphasizes that the body is not merely an instrument through which work is performed, but also a central site through which work is experienced, interpreted, and endured (Lawrence et al., 2023; Wolkowitz, 2006). In labour-intensive service environments, the body becomes a site of speed, endurance, discipline, emotional regulation, and accumulated fatigue (Farrugia et al., 2025; Kurkowska-Budzan & Galasińska, 2025). This perspective is especially relevant in professional kitchens, where workers operate under conditions of heat, continuous movement, prolonged standing, and repetitive physical strain (Kini et al., 2025; Melaku et al., 2024). In such settings, bodily exhaustion often extends beyond formal working hours and shapes workers’ recovery, endurance, and everyday functioning. The body therefore emerges not as an external element of employee experience (EX), but as one of its central experiential sites.

At the same time, labour in professional kitchens is organized through highly demanding temporal regimes (Kini et al., 2025). Time poverty is commonly associated with insufficient time for rest, family life, and personal activities (Perlow, 1999). However, in labour-intensive and service-driven environments, time poverty involves more than a quantitative lack of leisure; it also reflects a loss of control over time and a reduced capacity to organize everyday life according to one’s own priorities (Backhaus, 2022; Goodin et al., 2008). In professional kitchens, long shifts, unpredictable workloads, rotating schedules, and uncertain end-of-shift times reorganize everyday life around operational demands (Pickles et al., 2025; Seyitoğlu et al., 2023). As a result, workers experience not only limited time for recovery and social participation, but also a broader erosion of temporal autonomy. Time poverty therefore emerges as an experiential and psychosocial condition shaping how workers plan, interpret, and negotiate everyday life beyond work (Backhaus, 2022).

The pressures associated with professional kitchen labour also extend into domains traditionally positioned outside the workplace. Research on social reproduction and care labour has long emphasized that paid work and life beyond work cannot be treated as separate spheres (Kotiswaran, 2023; Rodríguez-Modroño et al., 2024). Activities such as caregiving, domestic organization, emotional support, and everyday maintenance are not external to labour, but are closely entangled with it (Murtola & Vallelly, 2023). In professional kitchens, long and irregular working hours, accumulated fatigue, and restricted recovery time often intensify tensions surrounding family responsibilities, social participation, and everyday care obligations (Minuzzo et al., 2026; Onur & Demirel, 2024). Consequently, care burdens and non-work responsibilities should not be understood as peripheral to EX, but as constitutive dimensions shaping how work is experienced emotionally, relationally, and socially.

Although the EX literature has developed significantly in recent years, much of it has conceptualized EX primarily through organizational touchpoints, managerial practices, workplace design, and individual attitudes (Andrés Reina et al., 2024; Itam & Ghosh, 2020; Morgan, 2017; Panneerselvam & Balaraman, 2022; Plaskoff, 2017). While this perspective has generated important insights, it remains limited in explaining labour-intensive environments in which work is experienced simultaneously through bodily exhaustion, temporal pressures, and responsibilities extending beyond the workplace. Existing research on embodied labour, time poverty, and social reproduction has illuminated these dimensions separately, yet these studies have largely developed in parallel. As a result, limited attention has been paid to how EX in labour-intensive settings is constituted through the cumulative and mutually reinforcing interaction of embodied strain, temporal loss of control, and non-work care burdens.

Another important yet underexplored dimension concerns the interpretive mechanisms through which demanding working conditions are normalized and rendered meaningful. In professional kitchens, the discourse of passion frequently frames hardship as an expected and legitimate aspect of professional identity. While passion may foster commitment and attachment to work, it may also normalize long working hours, bodily exhaustion, and broader psychosocial costs (Cech, 2021; Umney, 2025). In this sense, passion operates as a meaning-making mechanism through which demanding labour conditions become accepted and less visible (Tokumitsu, 2015).

Addressing this gap, the present study examines how EX is constituted in labour-intensive professional kitchen work at the intersection of embodied strain, time poverty, and care responsibilities. More specifically, the study explores how these pressures are experienced, interpreted, and negotiated within everyday work practices, and how the discourse of passion shapes the meaning and normalization of these experiences. Accordingly, the study addresses the following research questions:

**RQ1.**  *How do professional kitchen workers experience embodied strain, and how do they make sense of this strain within their everyday work practices?*

**RQ2.**  *How does time poverty shape professional kitchen workers’ relationship with work and their overall experience?*

**RQ3.**  *In what ways do care responsibilities and off-work burdens intersect with this experience, and how do they affect everyday life?*

**RQ4.**  *How does the discourse of passion frame, interpret, and render certain aspects of this multilayered experience less visible?*

This study makes three contributions to the literature. First, it demonstrates that EX in labour-intensive settings cannot be adequately understood solely through workplace interactions, organizational practices, or managerial processes. Instead, the findings show that EX is constituted through the interconnected pressures of bodily exhaustion, temporal constraints, and responsibilities extending beyond the workplace. Second, the study reconceptualizes EX as a life-embedded psychosocial process constituted across bodily, temporal, and extra-organizational domains simultaneously. Third, the study introduces two conceptual mechanisms—embodied time poverty and the hidden social burden of culinary labour—to explain how this life-embedded structure is produced and sustained in practice. In addition, it demonstrates how the discourse of passion operates as a meaning-making and normalizing mechanism that legitimizes demanding working conditions while obscuring their cumulative psychosocial costs.

## 2. Methods

### 2.1. Research Design

The study adopts a qualitative embedded single-case design focusing on the main kitchen of a five-star city hotel in Istanbul. A qualitative approach was considered appropriate because it enables the in-depth exploration of complex, multilayered, and context-dependent experiences while remaining sensitive to participants’ meanings and everyday realities (Creswell & Poth, 2018). In this respect, a case study design allows EX to be examined not only through individual perceptions but also in relation to the organizational, relational, and practical contexts in which it is produced and lived (Stake, 1995).

The bounded case in this study is the main kitchen of the selected hotel. This setting was treated as a single organizational case because it constitutes a clearly delimited work system with its own hierarchy, workflow, service rhythm, spatial organization, and occupational norms. The primary rationale for adopting a single-case design lies in the distinctive nature of professional kitchens as work environments characterized by intensive labour regimes, high physical demands, persistent time pressure, and strong normative expectations regarding endurance and commitment. These characteristics render the selected case analytically meaningful for an in-depth examination of how embodied, temporal, and social pressures are experienced, interpreted, and negotiated within a bounded organizational setting (Yin, 2018). Rather than aiming for statistical generalization, the study seeks to generate contextually grounded and theoretically meaningful insight into the psychosocial dynamics of EX in a labour-intensive work environment.

In this sense, the study follows the logic of analytical rather than statistical generalization. The aim is not to claim that the selected hotel kitchen represents all professional kitchens or all EX contexts, but to use a theoretically informative case to examine processes that may also be relevant to comparable labour-intensive service settings. The analytical transferability of the findings depends on the extent to which other contexts share similar boundary conditions, including high service intensity, hierarchical work organization, embodied labour demands, irregular and service-driven working hours, limited recovery opportunities, and strong occupational norms surrounding endurance, sacrifice, and passion. Accordingly, the findings should be read as theoretically transferable to similar professional kitchen contexts and comparable labour-intensive hospitality settings rather than universally generalizable across all forms of work.

The design is embedded because the study examines variation within the case across different roles, hierarchical positions, kitchen sections, shift arrangements, and care responsibility profiles. In this sense, the embedded units of analysis are the participants’ situated experiences within different functional and hierarchical locations of the same kitchen system. These embedded units include managerial and supervisory roles, such as head chef, sous chef, and chef de partie, as well as operational and support roles, such as demi chef, commis, pastry staff, and stewards. This embedded structure enables the analysis of how EX varies in relation to job role, organizational position, work section, shift type, and personal responsibilities (Scholz & Tietje, 2002). The study thus treats the main kitchen as a single organizational case while attending to internal variation across subunits within it, thereby allowing a more nuanced understanding of how EX is shaped across different locations, roles, and lived conditions within the same work system.

### 2.2. Case Description

The case examined in this study is the main kitchen of a five-star city hotel located in Istanbul, Türkiye. The kitchen operates within a complex production structure that simultaneously supports multiple service formats. The coexistence of à la carte service, buffet operations, and banquet production creates a work setting in which culinary labour unfolds under conditions requiring sustained pace, coordination, responsiveness, and flexibility. This multi-service structure generates fluctuations in workload both throughout the day and across service periods, exposing workers to intense and variable demands in physical, temporal, and organizational terms.

At the same time, the hierarchical organization of the kitchen, the distribution of tasks across sections, and the continuous nature of production contribute to patterned differences in workers’ everyday experiences across positions and responsibilities. Employees working in different roles are exposed to shared organizational pressures, yet they also encounter these pressures in distinct ways depending on their place within the labour process, their degree of responsibility, and the demands attached to their specific work areas.

In this respect, the selected case provides an analytically rich and revealing context for examining how EX is shaped through the intersection of embodied strain, time poverty, and care responsibilities. Its complex workflow, high service intensity, and internal differentiation make it particularly suitable for exploring how these pressures are produced, experienced, and negotiated within a bounded organizational environment.

### 2.3. Sample

The sample consists of 20 participants selected through a purposive sampling strategy from a total of 32 workers employed in the main kitchen of the hotel under study. Consistent with the qualitative research design, the aim of participant selection was not statistical representativeness, but rather to ensure sufficient diversity of experiences capable of providing rich, contextually grounded, and analytically meaningful insights into the research questions (Patton, 2015). Accordingly, the sample was constructed to capture variation across roles, responsibilities, and working conditions within the kitchen (see Table 1).

Participant selection was guided by the aim of capturing maximum variation within the bounded case. The researchers sought to include participants across hierarchical levels, kitchen sections, shift types, length of professional experience, gender, and care responsibility status. In this process, both managerial and supervisory roles, such as the head chef, sous chef, junior sous chef, and chef de partie, and operational and support roles, such as demi chefs, commis, pastry staff, and stewards, were included. Attention was also paid to representing different kitchen sections, including the hot kitchen, cold kitchen, banquet kitchen, à la carte kitchen, pastry section, breakfast section, butchery section, staff kitchen, and dishwashing area. In addition, participants with and without care responsibilities were deliberately included in order to examine how non-work responsibilities intersect with embodied strain and time poverty. Although the gender distribution of the kitchen workforce limited the number of female participants, women from different positions and sections were included to support variation in the analysis of care responsibilities and work–life experiences.

Data collection continued until conceptual saturation was reached, that is, the point at which no new themes or analytically relevant insights emerged from the data (Guest et al., 2006). Saturation was assessed during the ongoing data collection and analysis process by comparing each new interview with the emerging codes and candidate themes. As the later interviews began to confirm rather than substantially extend the developing thematic structure, the sample was considered sufficient to support a comprehensive and theoretically meaningful understanding of the phenomena under study (Saunders et al., 2018).

### 2.4. Data Collection

Data were collected primarily through semi-structured in-depth interviews. This method was considered particularly suitable because it enabled participants to articulate their experiences of working in professional kitchens in their own terms, while also allowing the systematic exploration of themes central to the study, including embodied strain, time poverty, care responsibilities, and the discourse of passion (Kallio et al., 2016; Kvale & Brinkmann, 2015). In line with the qualitative and interpretive orientation of the study, the interview format provided access not only to participants’ descriptions of working conditions, but also to how they experienced, interpreted, and made sense of these conditions in their everyday lives.

The interview guide was developed in line with the conceptual framework derived from the literature on embodied labour, time poverty, care burdens, and the discourse of passion. It consisted of open-ended questions designed to elicit in-depth accounts of participants’ everyday work practices, working conditions, use of time, experiences of fatigue and recovery, and responsibilities outside work across these dimensions (see Appendix A). In this way, the interview structure supported both consistency across interviews and sufficient flexibility for participants to elaborate on issues that were particularly salient in their own experiences (Kallio et al., 2016).

Interviews were conducted between 20 January 2026 and 13 February 2026. Depending on participants’ availability, they took place either in a quiet and private area within the workplace, outside the workplace, or online. The interviews were conducted in Turkish by the research team, whose members had prior experience in qualitative interviewing. Each interview lasted approximately 45 to 60 min. Before each interview, participants were informed about the purpose of the study, the voluntary nature of participation, their right to withdraw at any stage, and the confidentiality of their responses. Written or verbal informed consent was obtained before the interviews began.

With participants’ consent, all interviews were audio-recorded and subsequently transcribed verbatim in Turkish. The transcripts were checked against the audio recordings to ensure accuracy and to preserve the meaning, nuance, and experiential depth of participants’ accounts. During transcription and analysis, identifying information, such as names, specific personal details, and any potentially traceable references, was removed. Participants were assigned anonymized codes, and these codes were used consistently throughout the analysis and reporting process.

The study was approved by the Beykoz University Scientific Research and Publication Ethics Board (Protocol Code: E-45152895-299-2600000905; Approval No: 01/17; Approval Date: 19 January 2026). Throughout the data collection process, ethical principles were observed, participants’ identities were kept confidential, and anonymized codes were used during analysis (Orb et al., 2001).

To support and contextualize the interview data, field observations were conducted where possible, and notes were taken on workflow, pace, physical conditions, and the general organization of the kitchen environment. In addition, secondary materials such as shift schedules and work plans were reviewed to obtain contextual information relevant to the interpretation of participants’ narratives. Rather than being treated as direct units of analysis, these materials were used to enrich contextual understanding and to support the interpretation of the interview findings within the broader organizational setting (Merriam & Tisdell, 2016).

### 2.5. Data Analysis

The data were analyzed using a reflexive thematic analysis approach (Braun & Clarke, 2021). This method was selected because of its capacity to attend to participants’ meaning-making processes while also allowing for a flexible yet systematic examination of patterns across the dataset. It was particularly appropriate for the purposes of this study, as the research sought not only to identify recurring experiences but also to interpret how embodied strain, time poverty, and care responsibilities were experienced, understood, and articulated by participants within the context of professional kitchen work. Throughout the analysis, the conceptual framework guiding the study—aimed at capturing the embodied, temporal, and social dimensions of EX—provided an initial analytical orientation. However, coding was not restricted to pre-defined categories and remained open to meanings emerging from the data. In this sense, the study adopts an abductive analytical logic that integrates both inductive and deductive sensibilities, moving iteratively between empirical material and conceptual interpretation (Timmermans & Tavory, 2012). In this abductive process, EX served as the central conceptual frame, while embodied labour, time poverty, and social reproduction were used as sensitizing concepts to interpret how work was lived across bodily, temporal, and non-work domains.

The analysis proceeded through multiple stages. First, interview recordings were transcribed verbatim and read repeatedly by the researchers to ensure close familiarity with the data. This phase involved immersion in the dataset and careful attention to both explicit content and the broader experiential meanings embedded in participants’ accounts. Second, meaningful data segments were identified, and initial open codes were generated. During this coding process, particular attention was paid to participants’ accounts of embodied strain, uses of time, work–life relations, care responsibilities, and the ways in which these experiences were interpreted and normalized. Third, codes with similar content were clustered into higher-order categories, and themes were developed by examining patterns of convergence, divergence, and interrelation across these categories (Braun & Clarke, 2006).

To strengthen the transparency of the analytic process, coding was conducted in a structured but reflexive manner. Initial coding was first carried out manually using transcript files and analytic memos rather than qualitative data analysis software. This decision was consistent with the interpretive and reflexive orientation of the study, as the analysis required close engagement with the language, context, and experiential meanings of participants’ accounts. After initial codes were generated, the researchers compared coding notes, discussed differences in interpretation, and refined the code list through collaborative analytic meetings. Disagreements were not treated as reliability problems to be statistically resolved, but as opportunities for reflexive discussion and deeper interpretation, in line with the logic of reflexive thematic analysis.

Theme development was treated as an interpretive and iterative process rather than a purely mechanical one (Braun & Clarke, 2021). As analysis progressed, codes and candidate themes were repeatedly reviewed, refined, and reorganized in relation to both the dataset and the study’s conceptual concerns. The relationship between codes, higher-order categories, and themes was documented through analytic memos and theme-mapping tables. This iterative movement enabled the researchers to identify not only recurring topics but also the deeper structures of meaning through which participants made sense of their work and everyday lives. In this respect, the themes were constructed as analytically meaningful patterns that captured both shared experiences and the relational dynamics linking body, time, and non-work responsibilities.

Finally, the resulting themes were interpreted in relation to the study’s conceptual framework. Particular emphasis was placed on how embodied strain, time poverty, and care burdens intersect within EX, as well as on how the discourse of passion frames, legitimizes, and renders aspects of this experience less visible. In line with the principles of reflexive thematic analysis, the interpretive role of the researchers was explicitly acknowledged, and the themes were connected to broader theoretical discussions rather than being confined to a purely descriptive level (Terry et al., 2017).

Researcher reflexivity was also considered throughout the research process. One member of the research team had nearly ten years of practical experience in the field of gastronomy and culinary arts, which provided contextual sensitivity to kitchen work, occupational routines, and professional terminology. However, none of the researchers had any employment, managerial, ownership, friendship, or prior personal relationship with the hotel or with the participants included in the sample. This position was explicitly reflected upon during data collection and analysis in order to benefit from contextual familiarity while minimizing the risk of over-identification with the field.

### 2.6. Trustworthiness and Validity

In this study, multiple strategies were employed to enhance the trustworthiness and validity of the qualitative findings. First, triangulation was applied through the diversification of data sources (Denzin, 1978). Data obtained from semi-structured interviews were complemented by field observations and secondary materials such as shift schedules and work plans. Rather than being treated as direct units of analysis, these materials were used to enrich contextual understanding, support the interpretation of participants’ accounts, and situate EX within the broader organizational conditions in which it was produced. This approach helped prevent the findings from being reduced solely to interview narratives and supported a more comprehensive and context-sensitive analytical process (Patton, 2015).

The study also placed strong emphasis on reflexivity, with careful attention to the potential influence of researcher subjectivity on data generation and interpretation (Finlay, 2002). As noted in the data analysis section, one member of the research team had prior practical experience in gastronomy and culinary arts, while none of the researchers had any employment, managerial, ownership, friendship, or prior personal relationship with the hotel or the participants. This positionality was explicitly reflected upon during coding, theme development, and interpretation. The researchers used analytic memos and team discussions to examine how their assumptions, conceptual orientations, and field familiarity might shape the reading of the data. In this respect, the process of analysis was conducted not only descriptively, but also in a critical and reflexive manner (Berger, 2015).

Finally, the study adopted a thick description approach (Geertz, 1973). By presenting participants’ experiences, contextual features, and working conditions in detail, the study aimed to enable readers to develop an in-depth understanding of the research setting and the conditions under which the findings emerged. In doing so, it strengthens the analytical transferability of the findings to other contexts by providing sufficient contextual depth for readers to assess the relevance of the findings beyond the immediate case (Lincoln & Guba, 1985).

## 3. Findings

The findings of this study are organized into five main themes, derived from codes generated through the systematic analysis of participants’ narratives and the sub-themes constructed from these codes. Developed through reflexive thematic analysis, this thematic structure draws on recurring patterns and shared meanings within the data to provide a holistic account of the embodied, temporal, and social dimensions of EX in professional kitchens.

The distribution of findings across main themes, sub-themes, and codes is presented in Table 2. The following subsections examine each theme in detail, drawing on participants’ narratives and relating the findings to the study’s conceptual framework.

### 3.1. Main Theme 1: Embodied Strain and Physical Exhaustion

#### 3.1.1. Continuous Physical Demands

Participants described professional kitchen work as physically intense, continuous, and highly demanding. In particular, workers in hot kitchen, banquet, and à la carte sections emphasized the high pace, uninterrupted movement, and prolonged standing required by the job, describing physical demand as a fundamental component of their everyday work experience.

A participant working in the hot kitchen (K7, Commis, 3 years of experience) described this condition as follows: “Once the service starts, there’s no such thing as stopping. You’re constantly in motion. It’s already hot, and when you add pace, your body just can’t keep up after a while.” Similarly, a participant working in the banquet kitchen (K9, Commis, 4 years of experience) emphasized how physical demands increase during peak periods: “If there’s a wedding or a big event, the pace doubles. You work for hours without stopping.”

While participants in higher positions also acknowledged the intensity of physical demands, they tended to frame it as an inherent feature of the job. A Chef de Partie working in the hot kitchen (K3, 8 years of experience) stated: “That’s just the nature of this job. You’re on your feet, there’s heat, there’s pace.” Similarly, a participant in a more senior role (K19, Jr. Sous Chef, 10 years of experience) highlighted how physical demands are unevenly distributed across hierarchical positions: “Everyone works at the same pace, but those at lower levels feel it more because they’re constantly in the middle of the work.”

#### 3.1.2. Physical Wear and Exhaustion

Participants described the physical demands of professional kitchen work as cumulative and enduring rather than temporary. Chronic fatigue, bodily pain, loss of energy, and declining physical endurance emerged as recurring aspects of workers’ experiences, with exhaustion gradually becoming normalized through the ongoing continuity of work.

Lower-level workers, in particular, described physical exhaustion in more direct terms. A commis working in the hot kitchen (K7, 3 years of experience) expressed this as follows: “At first, it’s really hard, but then you get used to it. But getting used to it doesn’t mean you stop getting tired; it just means your body is constantly exhausted.” A more experienced participant (K14, Demi Chef, Hot Kitchen, 7 years of experience) described the gradual decline of physical endurance in the following terms: “It’s not like it used to be. As the years go by, your body can’t handle the same pace. The aches start, and the fatigue becomes permanent.”

Although middle- and upper-level participants expressed physical exhaustion in more controlled language, they also acknowledged its inevitability. A participant working as a Chef de Partie in the cold kitchen (K13, 6 years of experience) reflected on this as follows: “Fatigue is now part of the job. Sometimes it doesn’t go away even if you rest, and the next day you’re back in the same pace again.” Likewise, a participant in a more senior position (K19, Jr. Sous Chef, 10 years of experience) emphasized the ongoing nature of physical exhaustion: “In this job, fatigue is not temporary. There is a constant state of tiredness; you just learn how to manage it.”

It is also notable that physical wear is experienced in similar ways across different kitchen units. A participant working in the pastry section (K11, Pastry Chef, 9 years of experience) described it as follows: “At the end of the day, you’re not just tired; your body collapses. It’s not easy to recover and go back into the same pace the next day.”

#### 3.1.3. Bodily Continuity of Work

Participants described the embodied effects of professional kitchen work as extending beyond formal working hours. Post-work fatigue, insufficient recovery, and limited rest emerged as recurring experiences shaped by prolonged standing, high-paced production processes, limited break opportunities, and uninterrupted workflow.

These conditions were described in similar ways by participants working across different kitchen units. A participant working in the pastry section (K11, Pastry Chef, 9 years of experience) described the persistence of physical fatigue as follows: “At the end of the day, you’re not just tired; that fatigue stays with you. It doesn’t go away even before the next day begins.” Similarly, a participant working in the dishwashing area (K16, Steward, 4 years of experience) emphasized the inadequacy of recovery after work: “You go home, but you don’t feel rested. When you wake up, you’re still tired, as if you never stopped.”

While participants in higher positions also pointed to this continuity, they tended to frame it as inherent to the nature of the job. A participant working in the à la carte kitchen (K19, Jr. Sous Chef, 10 years of experience) expressed this as follows: “The shift ends, but it doesn’t really end. You leave physically, but your body is still in that pace.”

This continuity becomes even more pronounced when rest is fragmented or postponed. A participant working in the cold kitchen (K13, Chef de Partie, 6 years of experience) described this as follows: “You can’t really rest after work because there are other responsibilities at home. The fatigue stays with you—it never fully resets.”

### 3.2. Main Theme 2: Fragmentation of Time and Time Poverty

#### 3.2.1. Long and Irregular Working Hours

Participants described working hours in professional kitchens as long, irregular, and difficult to predict. Long shifts, variable schedules, and routinized overtime emerged as central features shaping workers’ experience of time. Participants associated these conditions not only with staff shortages and workload intensity, but also with service demands, last-minute operational changes, and the expectation that shifts continue until the work is completed.

Participants working in banquet and à la carte kitchens, in particular, emphasized that workload can escalate suddenly depending on events and customer volume. A participant working in the banquet kitchen (K4, Chef de Partie, 10 years of experience) described this situation as follows: “You never know when you’ll leave. You can’t leave until the work is done, so the idea of hours disappears.” A participant working in the à la carte kitchen (K5, Demi Chef, 6 years of experience) highlighted the gap between formal shift schedules and actual working time: “Normally the shift is set, but if the work runs late, you run late too. There’s an end time on paper, but not in practice.”

Lower-level workers described the effects of long and irregular working hours on everyday life more directly. A participant working in the cold kitchen (K8, Commis, 2 years of experience) expressed this as follows: “You know what time you’ll start in the morning, but not what time you’ll leave. You can’t make plans because it can change every day.” Similarly, a participant working in the pastry section (K12, Pastry Assistant, 5 years of experience) described the normalization of long working hours in these terms: “Working long hours is normal here. No one says, ‘Let’s leave early today.’ You stay until the work is finished.”

Participants in more senior positions, by contrast, tended to interpret this condition through the lens of operational necessity. A participant working in the main kitchen (K2, Sous Chef, 12 years of experience) offered the following reflection: “In this job, the idea of fixed hours is a bit flexible. Since it’s a service sector job, if there are customers, there’s work—so the hours can stretch.”

#### 3.2.2. Loss of Control over Time

Participants described time in professional kitchens as largely shaped by workflow demands rather than personal control. Difficulties in planning daily life, uncertainty surrounding rest time, and the continual reorganization of personal time around service intensity emerged as central dimensions of employee experience.

Shift workers, in particular, frequently referred to the uncertainty created by being unable to plan everyday life in advance. A participant working in the cold kitchen (K13, Chef de Partie, 6 years of experience) described this situation as follows: “I don’t even know when I’ll be free the next day. You can’t make plans because work can change at any moment.” Similarly, a participant working in the dishwashing area (K17, Steward, 3 years of experience) drew attention to the uncertainty of rest time: “It’s not even clear when you’ll get to rest. It depends on how busy it is—sometimes you don’t get to rest at all.”

Middle-level workers described the loss of control over time as a more structural problem. A participant working in the à la carte kitchen (K5, Demi Chef, 6 years of experience) expressed this as follows: “Time doesn’t belong to you here. You move according to how the work is going.” A participant working in the butchery section (K20, Chef de Partie, 8 years of experience) described the determination of time by work in these terms: “You don’t make the schedule—the schedule makes you. Whatever the work says, that’s what happens.”

Participants in more senior positions, by contrast, tended to interpret this condition in terms of operational necessity. A participant working in the main kitchen (K1, Head Chef, 18 years of experience) offered the following reflection: “In the kitchen, time isn’t planned; it’s managed. But even that management mostly depends on how intense things are.”

#### 3.2.3. Deprivation of Social and Personal Time

Participants frequently described professional kitchen work as limiting access to social and personal time. Difficulties participating in social activities, reduced personal time, and the weakening of social relationships emerged as visible consequences of time poverty, shaped by long working hours, unpredictable schedules, weekend and peak-period work, and the persistent expectation of availability.

Shift workers, in particular, expressed this disconnection from social life most clearly. A participant working in the à la carte kitchen (K10, Commis, 3 years of experience) described this situation as follows: “Your friends make plans, but you usually can’t join them. Because either you’re working, or you feel like you’re about to be working.” Another participant working in the hot kitchen (K3, Chef de Partie, 8 years of experience) described growing distance from social activities in the following terms: “Everyone goes out on the weekend, and you’re working. After a while, people stop inviting you anyway.”

Lack of access to personal time was another dimension frequently emphasized by participants. A participant working in the pastry section (K12, Pastry Assistant, 5 years of experience) expressed this as follows: “You don’t really have time set aside for yourself. You’re either working, or trying to rest.” Similarly, a participant working in the staff kitchen (K18, Chef de Partie, 8 years of experience) drew attention to the qualitative dimension of personal time: “Even if you do have free time, you don’t have the energy to do anything. So in a way, you have time, but you can’t use it.”

Among participants with care responsibilities, deprivation of social and personal time became an even more pronounced experience. A participant working in the breakfast section (K15, Chef de Partie, 7 years of experience) described this as follows: “After work, there are already other responsibilities waiting. You don’t have any time left for yourself. There’s no such thing as a social life.”

### 3.3. Main Theme 3: Carrying Non-Work Burdens and Care Responsibilities

#### 3.3.1. Pressure of Care Burdens and Work–Life Tension

Participants described professional kitchen work as placing significant pressure on care responsibilities and everyday life roles. Long and irregular working hours, variable shifts, early-starting or late-ending workdays, and limited recovery time made it difficult to sustain childcare and family responsibilities, with participants frequently framing this experience in terms of role conflict and emotional burden.

Participants with care responsibilities stated that the continuity and intensity demanded by the job often prevent them from adequately fulfilling family-related responsibilities. A participant working in the cold kitchen (K13, Chef de Partie, 6 years of experience) described this situation as follows: “If you have a child, this job is harder. You can’t always be there, and you miss a lot of things.” Another participant working on the day shift (K15, Chef de Partie, Breakfast Section, 7 years of experience) described the continuity between work and home in the following terms: “Work ends, but then another responsibility starts at home. It becomes impossible to separate the two.”

Participants also emphasized that, over time, this condition turns into an explicit conflict between work and home roles. A participant working in the à la carte kitchen (K5, Demi Chef, 6 years of experience) described this tension as follows: “Where there is work, private life stays in the background. It’s hard to carry both at the same time.” Likewise, a participant working in the butchery section (K20, Chef de Partie, 8 years of experience) described the difficulty of sustaining family roles in these terms: “You’re at work at the very times when you should be taking care of your family. That inevitably creates problems.”

Over time, this conflict also acquires an emotional dimension, generating feelings of guilt and inadequacy among workers. A participant working in the banquet kitchen (K4, Chef de Partie, 10 years of experience) expressed this as follows: “Sometimes you feel bad. You’re at work when you should be at home.” A younger participant (K8, Commis, 2 years of experience) described this emotional tension in the following terms: “You feel like you’re never fully enough anywhere.”

#### 3.3.2. Reorganization of Everyday Life by Work

Participants described professional kitchen work as directly shaping the organization of everyday life. The allocation of domestic time to recovery, the postponement of life outside work, and the restructuring of daily routines around work demands emerged as central features of this experience, particularly under conditions of irregular shifts, long workdays, limited rest opportunities, and the need to organize life around the pace of the following day.

The allocation of post-work time to rest and recovery was a recurring theme in participants’ accounts. A participant working in the main kitchen (K1, Head Chef, 18 years of experience) described this situation as follows: “After work, you don’t really have the energy to do anything else. You spend the time you have left resting.” Similarly, a participant working in the pastry section (K11, Pastry Chef, 9 years of experience) described the nature of post-work time in the following terms: “It looks like you have free time, but in reality, you use it to recover.”

Participants also stated that life outside work is often postponed and that everyday plans are shaped around the rhythm of work. A participant working in the cold kitchen (K8, Commis, 2 years of experience) expressed this as follows: “Even when you make plans, you think around work. You arrange everything according to it.”

Among workers with care responsibilities, this reorganization becomes even more pronounced. A participant working in the breakfast section (K15, Chef de Partie, 7 years of experience) described this situation as follows: “When you get home, you still arrange everything around work. You try to rest, and at the same time you squeeze in your other responsibilities.”

### 3.4. Main Theme 4: The Discourse of Passion and Normalization

#### 3.4.1. Normalization of Hardship

Participants described the embodied and temporal pressures of professional kitchen work as gradually becoming normalized through everyday discursive practices. Expressions such as “you get used to it” framed hardship not as an exceptional condition, but as a natural and unavoidable part of the job. Participants emphasized that the difficulties of the work do not disappear over time; rather, workers gradually reinterpret and normalize them.

A participant working in the à la carte kitchen (K10, Commis, 3 years of experience) described this process as follows: “At first, it feels difficult, but everyone says the same thing: you’ll get used to it. And then you really do get used to it, but in fact the conditions don’t change.” A participant working in the staff kitchen (K18, Chef de Partie, 8 years of experience) described the incorporation of hardship into the nature of the job in the following terms: “If you work in a kitchen, you enter knowing it’s going to be hard. No one says it’s easy anyway.”

This process of normalization becomes more pronounced with increasing experience. More senior workers tended to interpret hardship not as an exceptional circumstance, but as part of professional belonging and competence. A participant working in the butchery section (K20, Chef de Partie, 8 years of experience) expressed this as follows: “Struggling is part of this job. If you’re not struggling, then maybe you’re not really doing this job.”

#### 3.4.2. Legitimation Through Passion

Participants described the discourse of passion as an important mechanism through which demanding working conditions in professional kitchens become legitimized. The idea that “if you love it, you endure it” frequently framed embodied strain, long working hours, and social burdens as acceptable expressions of individual commitment, dedication, and professional identity.

A participant working in the hot kitchen (K7, Commis, 3 years of experience) described this situation as follows: “If you love this job, you endure it. If you don’t, you can’t survive this pace.” Similarly, a participant working in the banquet kitchen (K4, Chef de Partie, 10 years of experience) described the valorization of sacrifice in the following terms: “This job is a bit about sacrifice. The more you give, the more you get back.”

The discourse of passion not only encourages the acceptance of hardship but also attaches emotional meaning to it. A participant working in the pastry section (K12, Pastry Assistant, 5 years of experience) described this emotional attachment as follows: “It’s hard, but when it’s a job you love, it feels different. You get tired, but you still want to do it.” A more senior participant (K2, Sous Chef, 12 years of experience) described kitchen work in terms of belonging and identity: “Kitchen work is a bit like a way of life. If you don’t love it, you wouldn’t enter this pace in the first place.”

#### 3.4.3. Mechanisms of Invisibility

Participants described intense embodied strain and long working hours in professional kitchens as gradually becoming normalized and rendered less visible. The downplaying of fatigue and the limited questioning of excessive working hours emerged as overlooked dimensions of EX, sustained through the collective normalization and everyday acceptance of demanding working conditions.

Lower-level workers, in particular, emphasized that fatigue is rarely articulated and is often not taken seriously. A participant working in the cold kitchen (K8, Commis, 2 years of experience) described this situation as follows: “Fatigue is already normal for everyone. No one says ‘I’m tired,’ because everyone is in the same situation.” A participant working in the dishwashing area (K17, Steward, 3 years of experience) described the everyday normalization of fatigue in the following terms: “You get tired, but that’s what’s expected in this job. No one sees it as a problem.”

Long working hours are similarly accepted without being questioned. A participant working in the pastry section (K11, Pastry Chef, 9 years of experience) expressed this as follows: “Working long hours is normal here. No one asks why it’s like this.”

Participants in more senior positions tended to explain this invisibilization by referring to the nature of the job itself. A participant working in the main kitchen (K1, Head Chef, 18 years of experience) offered the following reflection: “The kitchen is already an intense environment. Fatigue is part of it, so it’s not something people dwell on.”

### 3.5. Main Theme 5: Intersectional Domain: The Convergence of Body, Time, and Social Burdens

#### 3.5.1. Cumulative and Amplifying Effects

Participants described embodied strain, time poverty, and care responsibilities as mutually reinforcing pressures that intensified one another within everyday kitchen work. Fatigue complicated the fulfilment of care responsibilities, limited recovery time deepened physical exhaustion, and overlapping demands accumulated across work and non-work domains, producing cumulative psychosocial pressures on EX.

The interaction between physical fatigue and care responsibilities was articulated particularly clearly by participants. A participant working in the banquet kitchen (K4, Chef de Partie, 10 years of experience) described this situation as follows: “You’re already exhausted when you leave work, and then there are things to be done at home. That’s when everything feels harder.” Similarly, a participant with care responsibilities (K6, Demi Chef, Cold Kitchen, 7 years of experience) described this interaction in the following terms: “It’s harder to do anything when you’re tired. But the things you have to do don’t change.”

Time poverty emerges as another factor that deepens this pressure. A participant working in the à la carte kitchen (K19, Jr. Sous Chef, 10 years of experience) described the relationship between time and fatigue as follows: “When you don’t have time, you can’t rest. And when you can’t rest, the fatigue accumulates.” A participant working in the staff kitchen (K18, Chef de Partie, 8 years of experience) described the layering of different burdens in the following terms: “When the fatigue from work, the lack of time, and responsibilities at home come together, everything feels heavier.”

#### 3.5.2. Organization of Life Around Work

Participants described professional kitchen work as organizing the broader structure of everyday life. The reconfiguration of routines around work, the erosion of work–life boundaries, and the growing centrality of work emerged as defining features of EX, shaped by long and irregular hours, shift arrangements, and the need to organize both rest and non-work activities around the rhythm of the job.

Participants working day shifts noted that, although their lives could be planned somewhat more predictably, those plans remained largely work-centered. A participant working in the pastry section (K11, Pastry Chef, 9 years of experience) described this situation as follows: “You build your life around it. How the day unfolds is determined by work.” A participant working in the main kitchen (K1, Head Chef, 18 years of experience) expressed this in the following terms: “In this job, life is shaped around the kitchen. You live according to it.”

For shift workers, by contrast, this condition produces a more fragmented and uncertain organization of life. A participant working in the cold kitchen (K6, Demi Chef, 7 years of experience) described the erosion of work–life boundaries as follows: “It’s not clear when it’s work and when it’s private life. The two are constantly blending into each other.”

Lower-level workers expressed this process in a more direct and encompassing way. A participant working in the hot kitchen (K7, Commis, 3 years of experience) described the centrality of work as follows: “Most of your life is spent on work. You already live according to it.”

#### 3.5.3. Reproduced Cycle of Labour

Participants described embodied strain, time poverty, and social burdens as part of a recurring labour cycle in which exhaustion and demanding working conditions were continually reproduced. The normalization of hardship, limited expectations for change, and repeated cycles of fatigue and partial recovery emerged as defining features of this experience.

Middle-level workers articulated this cyclical structure particularly clearly. A participant working in the à la carte kitchen (K5, Demi Chef, 6 years of experience) described this situation as follows: “There’s always the same cycle. Intensity, fatigue, then a bit of recovery—but then it starts all over again.” A participant working in the hot kitchen (K14, Demi Chef, 7 years of experience) described the limited possibility of change in the following terms: “You don’t think things will change. Because it has always been like this, and it just keeps going like this.”

Lower-level workers tended to describe this cycle more directly through the repeated experience of exhaustion. A participant working in the banquet kitchen (K9, Commis, 4 years of experience) expressed this as follows: “You get tired, you try to rest, but then the same pace starts again. It feels like it never ends.” Similarly, a participant working in the butchery section (K20, Chef de Partie, 8 years of experience) described this cyclical structure in the following terms: “After a while, you get used to it—but that’s not a good thing. Because it means you’ve accepted that it won’t change.”

Participants in more senior positions, by contrast, tended to explain this condition by referring to the nature of the sector and the job itself. A participant working in the main kitchen (K2, Sous Chef, 12 years of experience) offered the following reflection: “This sector has always been like this. Intensity, pace, fatigue… these things don’t change.”

#### 3.5.4. Integrated Employee Experience

Participants described EX in professional kitchens as an integrated experience in which embodied strain, time poverty, and social burdens were deeply intertwined. Workers frequently portrayed the boundaries between body, time, work, and social life as difficult to sustain, emphasizing that EX was shaped not only through workplace interactions, but also through the extension of work into broader domains of everyday life.

This integrated structure is clearly visible in participants’ accounts. A participant working in the à la carte kitchen (K5, Demi Chef, 6 years of experience) described this situation as follows: “This job is not just a job. It affects your time, your energy, your life. Everything is connected.” A participant working in the pastry section (K12, Pastry Assistant, 5 years of experience) articulated this integrated structure in the following terms: “There is fatigue, there’s no time, and then there are responsibilities outside work. It all happens at once.”

Participants in more senior positions also referred to this integration, though in broader terms. A participant working in the main kitchen (K1, Head Chef, 18 years of experience) described this condition as follows: “If you work in a kitchen, your life is shaped around it too. It’s not easy to separate work from life.” Similarly, a participant working in the cold kitchen (K6, Demi Chef, 7 years of experience) expressed this more directly: “Work, time, and life get mixed together. You can’t think about them separately.”

## 4. Discussion

### 4.1. From Workplace-Centred EX to Life-Embedded EX

The findings of this study suggest that EX in labour-intensive professional kitchen work cannot be adequately understood solely through organizational touchpoints, managerial practices, or workplace interactions. Rather, the findings demonstrate that EX is constituted through the cumulative and mutually reinforcing interaction of embodied strain, temporal constraints, care responsibilities, and the interpretive meanings attached to work. In this respect, the study moves beyond dominant workplace-centred approaches to EX and proposes a reconceptualization of EX as a life-embedded psychosocial process.

Existing EX literature has made important contributions by demonstrating how EX work through organizational culture, leadership practices, workplace design, and everyday organizational interactions (Morgan, 2017; Plaskoff, 2017). More recent studies have further emphasized the strategic importance of EX for organizational engagement, performance, and well-being (Andrés Reina et al., 2024; Itam & Ghosh, 2020; Panneerselvam & Balaraman, 2022). However, these approaches have largely conceptualized EX within the boundaries of the workplace itself. By contrast, the present findings suggest that, in labour-intensive settings such as professional kitchens, EX extends beyond organizational space and becomes intertwined with bodily exhaustion, the erosion of temporal autonomy, and responsibilities embedded in everyday life.

This theoretical shift becomes visible particularly through participants’ accounts of fatigue, recovery, and the organization of life around work. The findings show that bodily exhaustion does not end with the formal completion of the shift; rather, fatigue continues to shape workers’ recovery, social participation, care responsibilities, and everyday functioning beyond work hours. In this respect, the study extends the literature on embodied labour, which has long emphasized that the body is not merely a passive instrument of work, but a constitutive site through which labour is experienced and endured (Warhurst & Nickson, 2007; Wolkowitz, 2006; Zampoukos, 2021). The findings further demonstrate that the embodied effects of work persist beyond the workplace itself, transforming EX into a continuously lived psychosocial condition rather than a phenomenon limited to organizational encounters.

A similar pattern emerges in relation to time poverty. Existing research has often conceptualized time poverty primarily in terms of reduced leisure time or excessive working hours (Burchardt, 2008; Venn & Strazdins, 2017). While the present findings are broadly consistent with studies emphasizing work-time control and schedule unpredictability (Backhaus, 2022; Schoellbauer et al., 2022), they also suggest that time poverty operates as a broader psychosocial process. Participants’ narratives indicate that work reorganizes everyday life around operational rhythms, weakens perceived autonomy over time, and constrains workers’ ability to rest, plan, and sustain social participation. In this sense, time poverty emerges not merely as a temporal shortage, but as an experiential condition through which work extends its influence into broader domains of life (Kim et al., 2025; Ye et al., 2024).

The findings also highlight the importance of care responsibilities and social reproduction processes in shaping EX. Research on social reproduction and care labour has long demonstrated that paid work and domestic responsibilities are deeply interconnected rather than separate domains (Jones et al., 2024; Kotiswaran, 2023; Sinha et al., 2024). The present study extends this perspective by showing how care responsibilities become intertwined with embodied exhaustion and restricted recovery in professional kitchen work. Participants frequently described tensions surrounding childcare, domestic responsibilities, social participation, and emotional adequacy, suggesting that non-work burdens are not peripheral to EX but constitutive of how work is emotionally and relationally lived beyond the workplace.

Taken together, these findings demonstrate that EX in labour-intensive work is not simply shaped by organizational processes occurring within the workplace, but constituted across bodily, temporal, and extra-organizational domains simultaneously. The originality of this conceptualization therefore lies in repositioning EX itself as a life-embedded psychosocial process extending across the broader terrain of everyday life.

### 4.2. Embodied Time Poverty and the Hidden Social Burden of Culinary Labour

The findings of this study further suggest that the relationship between embodied strain, temporal constraints, and responsibilities beyond work is not incidental, but organized through interconnected psychosocial mechanisms that shape how EX is lived and sustained in labour-intensive work. Within this framework, two conceptual mechanisms emerge as particularly significant: embodied time poverty and the hidden social burden of culinary labour. Together, these mechanisms explain how work extends beyond the workplace and becomes embedded within bodily, temporal, and relational domains of everyday life.

The first mechanism, embodied time poverty, captures the idea that time scarcity in professional kitchens is experienced not only as a quantitative shortage of free time but also through bodily exhaustion, constrained recovery, and diminished autonomy over everyday life. Existing literature has extensively examined the effects of long working hours, work intensity, schedule unpredictability, and reduced work-time control on recovery and well-being (Backhaus, 2022; Baethge et al., 2026; Schoellbauer et al., 2022; Vieten et al., 2023). Similarly, studies on time poverty have generally emphasized the relationship between limited discretionary time and reduced quality of life (Burchardt, 2008; Venn & Strazdins, 2017). However, the present findings suggest that, in labour-intensive kitchen work, temporal pressure and bodily fatigue are not experienced as separate conditions. Rather, workers experience time scarcity through the body itself.

Participants’ narratives indicate that long and unpredictable shifts, continuous movement, and high operational pace reduce not only the amount of available time, but also workers’ physical and psychological capacity to use that time meaningfully. Even when time outside work formally exists, accumulated fatigue often transforms that time into a period devoted primarily to recovery rather than social participation, self-direction, or personal life. In this respect, embodied time poverty refers to a condition in which diminished control over time becomes inseparable from bodily exhaustion and constrained recovery. The findings therefore extend existing literature by showing that time poverty in labour-intensive work is simultaneously temporal, embodied, and psychosocial in nature.

The second mechanism, the hidden social burden of culinary labour, explains how the pressures generated by professional kitchen work extend into family life, care responsibilities, and broader social relations. Research on social reproduction and care labour has long emphasized that paid labour is sustained through everyday processes such as caregiving, domestic work, and emotional support (Bhattacharya, 2017; Folbre, 2006; Jones et al., 2024). However, these processes frequently remain marginal within organizational analyses of work and employee experience. The present findings suggest that, in professional kitchens, responsibilities outside work are not external to EX, but actively shape how work is emotionally and relationally experienced.

Participants frequently described tensions surrounding childcare, domestic responsibilities, social participation, and feelings of inadequacy arising from the limited time and energy left after work. In this sense, the effects of kitchen labour continue beyond the workplace by reorganizing everyday routines, constraining participation in social life, and intensifying emotional pressures associated with care responsibilities. These findings align with studies emphasizing the entanglement of paid labour and social reproduction processes (Kotiswaran, 2023; Sinha et al., 2024), while extending this literature by demonstrating how these pressures become integrated into the lived structure of EX itself. The hidden social burden of culinary labour therefore refers not only to practical responsibilities beyond work but also to the emotional, relational, and psychosocial pressures generated through the ongoing interaction between labour demands and everyday life obligations.

Taken together, these two mechanisms demonstrate that EX in labour-intensive professional kitchen work is produced through the cumulative interaction of bodily exhaustion, temporal loss of control, and social responsibilities extending beyond organizational boundaries. In this respect, embodied time poverty and the hidden social burden of culinary labour are not peripheral outcomes of work, but constitutive mechanisms through which life-embedded EX is continuously reproduced and sustained.

### 4.3. Passion as a Meaning-Making and Normalizing Mechanism

An additional contribution of the findings concerns the role of passion as a discursive and interpretive mechanism through which demanding working conditions are rendered meaningful, acceptable, and sustainable. Existing research has often emphasized the positive dimensions of passion, particularly its relationship with motivation, engagement, commitment, and professional identity (Ho et al., 2011; Vallerand et al., 2003). However, critical perspectives increasingly suggest that the discourse of passion may also obscure structural inequalities and normalize intensive labour conditions by framing hardship as an expression of individual dedication and self-fulfillment (Cech, 2021; Tokumitsu, 2015; Umney, 2025).

The findings of this study extend these critical perspectives within the context of professional kitchen work. Participants frequently interpreted long working hours, bodily exhaustion, and the sacrifices required outside work through expressions such as “you get used to it” and “if you love it, you endure it.” These narratives suggest that passion operates not merely as a personal emotional attachment to work but also as a meaning-making framework through which hardship becomes normalized and less open to critique. In this sense, difficult working conditions are repositioned less as structural or organizational problems and more as expected dimensions of professional commitment and occupational identity.

This interpretation is theoretically significant because it demonstrates that EX is shaped not only by objective working conditions but also by the interpretive vocabularies through which those conditions are justified, tolerated, and emotionally sustained. Passion therefore functions simultaneously as a motivational resource and as a normalizing mechanism that legitimizes bodily wear, temporal dispossession, and sacrifices extending into everyday life. Within labour-intensive professional kitchen work, the discourse of passion contributes to the reproduction of life-embedded EX by making the cumulative psychosocial costs of labour appear natural, meaningful, and professionally necessary.

**Figure 1:**
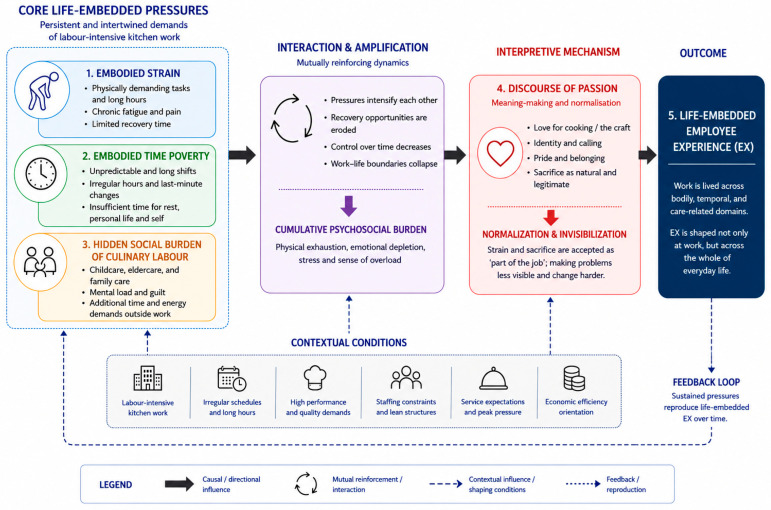
The life-embedded employee experience framework.

Figure 1 summarizes the conceptual framework developed from the findings of the study. As illustrated in the figure, embodied strain, embodied time poverty, and the hidden social burden of culinary labour operate as mutually reinforcing pressures in labour-intensive kitchen work. These pressures are interpreted and normalized through the discourse of passion, contributing to the reproduction of life-embedded EX across bodily, temporal, and everyday-life domains.

## 5. Conclusions and Implications

### 5.1. General Conclusion

This study shows that EX in professional kitchens is not confined to workplace interactions or task-related demands, but is constituted through the interconnected effects of bodily exhaustion, temporal constraints, care responsibilities, and the meanings attached to work. The findings demonstrate that professional kitchen work extends into everyday life by shaping workers’ recovery, perceived control over time, social participation, and capacity to sustain responsibilities beyond work.

The study’s main contribution is to reconceptualize EX as a life-embedded psychosocial process. Within this framework, embodied time poverty explains how time pressure becomes inseparable from bodily fatigue and limited recovery, while the hidden social burden of culinary labour makes visible how care and everyday life demands become constitutive elements of EX. The findings also show that the discourse of passion operates as a meaning-making and normalizing mechanism that legitimizes demanding working conditions and obscures their cumulative psychosocial costs. Together, these insights suggest that EX in labour-intensive work should be understood not only as an organizational phenomenon, but as a process lived across the body, time, and everyday life.

### 5.2. Practical and Policy Implications

The findings of this study suggest several organizational and policy-related implications for labour-intensive hospitality settings such as professional kitchens. At the organizational level, working time and shift systems should be designed in more predictable, equitable, and sustainable ways. Ensuring sufficient recovery time between shifts, limiting excessively fragmented schedules, and distributing high-intensity workloads more evenly may help reduce accumulated bodily exhaustion and support workers’ long-term well-being.

In addition, organizations should manage work intensity more carefully. Chronic understaffing, uneven workload distribution, and insufficient recovery opportunities intensify fatigue, time pressure, and psychosocial strain. Maintaining adequate staffing levels, balancing task allocation, and strengthening opportunities for rest and recovery during and between shifts are therefore important not only for operational continuity but also for sustaining employees’ physical and psychological functioning.

The findings further indicate that organizational approaches to EX should take greater account of responsibilities extending beyond the workplace. Care-sensitive scheduling practices, more flexible leave arrangements, and greater consideration of non-work demands may help employees manage tensions between work and everyday life more sustainably. In labour-intensive service environments, EX should therefore be approached not solely as a workplace issue, but as a broader organizational and social concern involving recovery, temporal autonomy, and everyday life sustainability.

At the policy level, the findings suggest the need for broader regulatory approaches addressing psychosocial as well as physical dimensions of work. Standards concerning minimum recovery periods between shifts, limits on unpredictable overtime, advance scheduling practices, and maximum consecutive working hours may help reduce chronic fatigue and temporal instability in labour-intensive hospitality work. Similarly, occupational health and labour inspection frameworks should address psychosocial risks such as time poverty, work–life disruption, and chronic exhaustion alongside traditional physical safety concerns.

Finally, the study highlights the importance of making care responsibilities more visible within labour and employment policy. Family-friendly work arrangements, care-sensitive scheduling standards, and support mechanisms for workers with childcare, eldercare, or dependent-care responsibilities may help reduce the cumulative embodied and temporal burdens identified in this study. More broadly, sustainable labour policy in hospitality should consider not only service quality and economic efficiency, but also the bodily, temporal, and social conditions through which labour is experienced and sustained.

### 5.3. Limitations

This study has several limitations. First, it is based on an embedded single-case study conducted in the main kitchen of a five-star city hotel in Istanbul. Although this context provides an analytically rich setting due to its high service standards, multi-service structure, hierarchical organization, and intense operational pace, it also limits the transferability of the findings to other types of kitchens, organizational scales, and national contexts. Therefore, the findings should not be interpreted as statistically representative of all professional kitchens or EX contexts. Rather, their analytical transferability is strongest for work settings that share similar boundary conditions, including labour-intensive service work, embodied job demands, irregular and service-driven working hours, limited recovery opportunities, hierarchical work organization, and occupational norms that valorize endurance, sacrifice, and passion.

Second, the findings were generated within a specific organizational and sectoral setting. The hierarchical structure, temporal regime, and professional norms of professional kitchens may have shaped participants’ narratives and the ways in which they interpreted and articulated their experiences. Accordingly, the patterns identified in this study reflect not only individual lived experiences but also the influence of the organizational culture and occupational discourses within which those experiences are embedded.

Third, although the study makes visible the importance of care responsibilities and non-work burdens, it does not examine in comparative detail how these dimensions vary according to gender, household structure, or class-based differences. Similarly, differences in experience across hierarchical positions could only be addressed to a limited extent. Future research would benefit from examining how these variations shape the experience, interpretation, and distribution of work-related pressures across different groups.

### 5.4. Directions for Future Research

Future research can make significant contributions to the literature by testing and extending the conceptual framework developed in this study across different contexts. First, comparative studies covering various types of kitchens—such as fine dining restaurants, chain restaurants, catering systems, and independent establishments—could more clearly reveal the conditions under which embodied strain, time poverty, and social burdens converge or diverge. Similarly, research conducted in different national and cultural contexts would be valuable for understanding how professional kitchen labour is shaped by local labour regimes and how these conditions influence the lived experience of work, recovery, and everyday life.

In addition, the ways in which EX varies across hierarchical positions could be examined more systematically. Studies comparing roles such as head chef, sous chef, chef de partie, commis, and support staff in terms of embodied strain, perceived control over time, and non-work burdens could make internal differentiations within kitchen labour more visible. Furthermore, research comparing the experiences of women and men—particularly in relation to care responsibilities and non-work burdens—could contribute to a more nuanced understanding of the gendered and relational dimensions of EX.

Third, the effects of different shift arrangements on EX should be addressed as a distinct line of inquiry. The impacts of day, night, and rotating shift systems on bodily recovery, participation in social life, the management of care responsibilities, and work–life tension could be examined more systematically. Such research would be particularly valuable for understanding how temporal structures shape well-being, perceived autonomy, and the sustainability of work over time.

Finally, quantitative and mixed-methods studies could test the conceptual framework proposed in this study across larger samples. In particular, research examining how the concepts of embodied time poverty, the hidden social burden of culinary labour, and life-embedded EX manifest across different sectors and occupational contexts would have strong potential to extend the theoretical contribution of this study. Such work could also explore the relationships between these constructs and key psychosocial outcomes such as well-being, recovery, job satisfaction, and long-term engagement with work.

## Figures and Tables

**Table 1:** Participant profile.

Participant Code	Position	Gender	Experience (Years)	Shift Type	Care Responsibility
K1	Head Chef (Main Kitchen)	Male	18	Day shift	Yes
K2	Sous Chef (Main Kitchen)	Male	12	Day shift	No
K3	Chef de Partie (Hot Kitchen)	Female	8	Day shift	Yes
K4	Chef de Partie (Banquet Kitchen)	Male	10	Rotating shifts	No
K5	Demi Chef (À la Carte Kitchen)	Male	6	Rotating shifts	No
K6	Demi Chef (Cold Kitchen)	Female	7	Day shift	Yes
K7	Commis (Hot Kitchen)	Male	3	Day shift	No
K8	Commis (Cold Kitchen)	Female	2	Rotating shifts	No
K9	Commis (Banquet Kitchen)	Male	4	Day shift	Yes
K10	Commis (À la Carte Kitchen)	Male	3	Rotating shifts	No
K11	Pastry Chef (Pastry Section)	Female	9	Day shift	Yes
K12	Pastry Assistant (Pastry Section)	Male	5	Rotating shifts	No
K13	Chef de Partie (Cold Kitchen)	Female	6	Day shift	Yes
K14	Demi Chef (Hot Kitchen)	Male	7	Rotating shifts	No
K15	Chef de Partie (Breakfast Section)	Female	7	Day shift	Yes
K16	Steward (Dishwashing Area)	Male	4	Rotating shifts	Yes
K17	Steward (Dishwashing Area)	Female	3	Day shift	No
K18	Chef de Partie (Staff Kitchen)	Male	8	Day shift	No
K19	Jr. Sous Chef (À la Carte Kitchen)	Male	10	Rotating shifts	No
K20	Chef de Partie (Butchery Section)	Male	8	Day shift	No

**Table 2:** Main themes, sub-themes, and codes.

Main Themes	Sub-Themes	Codes
Main Theme 1: Embodied Strain and Physical Exhaustion	1.1. Continuous Physical Demands	(i) Exposure to high heat (ii) Prolonged standing (iii) Intense and uninterrupted pace (iv) Pressure for physical speed
	1.2. Physical Wear and Exhaustion	(i) Chronic fatigue (ii) Bodily pain and strain (iii) Loss of energy (iv) Decline in physical endurance
	1.3. Bodily Continuity of Work	(i) Post-work fatigue (ii) Lack of recovery (iii) Insufficient rest
Main Theme 2: Fragmentation of Time and Time Poverty	2.1. Long and Irregular Working Hours	(i) Long shifts (ii) Unpredictable working hours (iii) Normalization of overtime
	2.2. Loss of Control over Time	(i) Inability to plan daily life (ii) Uncertainty of rest time (iii) Time determined by work
	2.3. Deprivation of Social and Personal Time	(i) Inability to participate in social activities (ii) Lack of access to personal time (iii) Weakening of social relationships
Main Theme 3: Carrying Non-Work Burdens and Care Responsibilities	3.1. Pressure of Care Burdens and Work–Life Tension	(i) Difficulties in childcare and family responsibilities (ii) Conflict between work and domestic roles (iii) Inability to sustain family roles (iv) Guilt and emotional exhaustion
	3.2. Reorganization of Everyday Life by Work	(i) Allocation of home time to recovery (ii) Postponement of life outside work (iii) Structuring daily routines around work
Main Theme 4: The Discourse of Passion and Normalization	4.1. Normalization of Hardship	(i) “You get used to it” discourse (ii) Viewing hardship as part of the job
	4.2. Legitimation through Passion	(i) “If you love it, you endure it” mindset (ii) Valuing sacrifice (iii) Emotional attachment to work
	4.3. Mechanisms of Invisibility	(i) Downplaying fatigue (ii) Lack of questioning of long working hours
Main Theme 5: Intersectional Domain: The Convergence of Body, Time, and Social Burdens	5.1. Cumulative and Amplifying Effects	(i) Fatigued body intensifying care burdens (ii) Time poverty exacerbating fatigue (iii) Overlapping demands
	5.2. Organization of Life around Work	(i) Reconfiguration of life routines around work (ii) Disappearance of work–life boundaries (iii) Work becoming the central organizing force of life
	5.3. Reproduced Cycle of Labour	(i) Transformation of hardships into a stable work regime (ii) Weak expectations of change (iii) Cyclical exhaustion
	5.4. Integrated Employee Experience	(i) Non-separation of body, time, and social burdens (ii) Work shaping all domains of life

## Data Availability

The data supporting the findings of this study, consisting of qualitative interview transcripts collected under conditions of strict confidentiality, are available from the corresponding author upon reasonable request and subject to appropriate ethical approval. Given the sensitive nature of these data and the need to protect participant privacy, full transcripts cannot be made publicly available. De-identified excerpts relevant to the analysis are included in the manuscript.

## Abbreviation

The following abbreviation is used in this manuscript:

EX	Employee experience

## Appendix A

1. Could you briefly describe your current role, area of responsibility in the kitchen, and your daily workflow?
2. How would you generally describe working in a professional kitchen?
3. What are the most physically demanding aspects of your job?
4. How would you evaluate the effects of this work on your body over time?
5. Do the physical effects of work continue after your shift ends? How do you experience this in your daily life?
6. How do you experience your working hours and shift arrangements?
7. To what extent do you feel you have control over your time?
8. How does your job affect your social life, your time for rest, and the time you can allocate to yourself?
9. Could you describe how your job affects your family life, domestic responsibilities, or care responsibilities?
10. How do you experience balancing work and responsibilities outside work?
11. In general, how does your job shape the organization of your everyday life?
12. How are the challenges of kitchen work typically discussed and interpreted among workers?
13. In your view, what keeps people working in this job, and what makes it bearable, meaningful, or valuable?
14. Do you think the difficulties of this job are sometimes seen as part of the “nature of the work” or the “requirements of the profession”? How do you evaluate this?
15. When you consider the effects of your job on your body, your time, and your responsibilities outside work together, what overall picture do you see?
16. In your opinion, what needs to change most to improve the experience of working in a professional kitchen?
17. Is there anything else you would like to add about your experiences or views?

## References

[B1-behavsci-16-00905] Andrés Reina M. P., Díaz-Muñoz R., Rodríguez-Fernández M. (2024). Employee experience: A systematic review. European Public & Social Innovation Review.

[B2-behavsci-16-00905] Angelini F., Castellani M., Pattitoni P. (2025). Evaluating chef’s creativity and restaurant quality: An empirical analysis of the role of gastronomic guides in the Italian fine-dining market. Kyklos.

[B3-behavsci-16-00905] Backhaus N. (2022). Working time control and variability in Europe revisited: Correlations with health, sleep, and well-being. International Journal of Environmental Research and Public Health.

[B4-behavsci-16-00905] Baethge A., Rigotti T., Mueller A. (2026). Working longer and more intensively to get the work done: Self-endangering work behaviour and its implications for health. Work & Stress.

[B5-behavsci-16-00905] Berger R. (2015). Now I see it, now I don’t: Researcher’s position and reflexivity in qualitative research. Qualitative Research.

[B6-behavsci-16-00905] Bhattacharya T. (2017). Social reproduction theory: Remapping class, recentering oppression.

[B7-behavsci-16-00905] Braun V., Clarke V. (2006). Using thematic analysis in psychology. Qualitative Research in Psychology.

[B8-behavsci-16-00905] Braun V., Clarke V. (2021). Thematic analysis: A practical guide.

[B9-behavsci-16-00905] Burchardt T. (2008). Time and income poverty *(CASE Report No. 57)*.

[B10-behavsci-16-00905] Cech E. A. (2021). The trouble with passion: How searching for fulfillment at work fosters inequality.

[B11-behavsci-16-00905] Cerasa A., Fabbricatore C., Ferraro G., Pozzulo R., Martino I., Liuzza M. T. (2020). Work-related stress among chefs: A predictive model of health complaints. Frontiers in Public Health.

[B12-behavsci-16-00905] Creswell J. W., Poth C. N. (2018). Qualitative inquiry and research design: Choosing among five approaches.

[B13-behavsci-16-00905] Denzin N. K. (1978). The research act: A theoretical introduction to sociological methods.

[B14-behavsci-16-00905] dos Anjos S. J. G., Kuhn V. R., Lima Manzi M. F. (2026). Culinary innovation from the perspective of restaurant and haute cuisine studies. Journal of Culinary Science & Technology.

[B15-behavsci-16-00905] Farrugia D., Coffey J., Gill R., Sharp M., Threadgold S. (2025). Youth and hospitality work: Skills, subjectivity and affective labour. Journal of Sociology.

[B16-behavsci-16-00905] Finlay L. (2002). “Outing” the researcher: The provenance, process, and practice of reflexivity. Qualitative Health Research.

[B17-behavsci-16-00905] Folbre N. (2006). Measuring care: Gender, empowerment, and the care economy. Journal of Human Development.

[B18-behavsci-16-00905] Geertz C. (1973). The interpretation of cultures.

[B19-behavsci-16-00905] Geyser I., Wakelin-Theron N., Esterhuyse N. (2023). The influence of work shifts on burnout for millennial chefs in the Western Cape, South Africa. Journal of Applied Sciences in Travel and Hospitality.

[B20-behavsci-16-00905] Goodin R. E., Rice J. M., Bittman M., Saunders P. (2008). Discretionary time: A new measure of freedom.

[B21-behavsci-16-00905] Guest G., Bunce A., Johnson L. (2006). How many interviews are enough? An experiment with data saturation and variability. Field Methods.

[B22-behavsci-16-00905] Ho V. T., Wong S.-S., Lee C. H. (2011). A tale of passion: Linking job passion and cognitive engagement to employee work performance. Journal of Management Studies.

[B23-behavsci-16-00905] Itam U., Ghosh N. (2020). Employee experience management: A new paradigm shift in HR thinking. International Journal of Human Capital and Information Technology Professionals.

[B24-behavsci-16-00905] Jones C., White L., Slater T., Pluquailec J. (2024). Hospitality work as social reproduction: Embodied and emotional labour during COVID-19. Sociology.

[B25-behavsci-16-00905] Kallio H., Pietilä A.-M., Johnson M., Kangasniemi M. (2016). Systematic methodological review: Developing a framework for a qualitative semi-structured interview guide. Journal of Advanced Nursing.

[B26-behavsci-16-00905] Kim D., Hu D., Harold C. M. (2025). Working around unpredictable clocks: Examining the impact of last-minute schedule changes on perceived contract breach and job performance. Human Relations.

[B27-behavsci-16-00905] Kini R. S., Dinesh T. K., Shetty A., K A. (2025). Exploring the risks faced by hotel kitchen professionals: From stovetop to safety. Cogent Social Sciences.

[B28-behavsci-16-00905] Kotiswaran P. (2023). Laws of social reproduction. Annual Review of Law and Social Science.

[B29-behavsci-16-00905] Kuhn V. R., dos Anjos S. J. G., Mundet L. (2025). Innovation and creativity in the restaurant sector: A systematic review of artifacts. Journal of Hospitality and Tourism Horizons.

[B30-behavsci-16-00905] Kurkowska-Budzan M., Galasińska A. (2025). Embodied labour: Interdisciplinary perspectives on work’s cultural heritage in modern Europe.

[B31-behavsci-16-00905] Kvale S., Brinkmann S. (2015). Interviews: Learning the craft of qualitative research interviewing.

[B32-behavsci-16-00905] Lawrence T. B., Schlindwein S., Casey C. (2023). Organizational body work: Efforts to shape human bodies in organizations. Academy of Management Annals.

[B33-behavsci-16-00905] Lincoln Y. S., Guba E. G. (1985). Naturalistic inquiry.

[B34-behavsci-16-00905] Melaku C., Abere G., Zele Y. T., Mamaye Y., Abebaw T., Bezie A. E., Tesfaye A. H., Worede E. A. (2024). Occupational heat exposure-related symptoms prevalence and associated factors among hospitality industry kitchen workers in Ethiopia: Wet bulb globe temperature. Safety and Health at Work.

[B35-behavsci-16-00905] Merriam S. B., Tisdell E. J. (2016). Qualitative research: A guide to design and implementation.

[B36-behavsci-16-00905] Minuzzo D. A., Kraemer F. B., Gracia-Arnaiz M. (2026). Gender relations in culinary work in restaurant kitchens: An integrative review of the literature. International Journal of Gastronomy and Food Science.

[B37-behavsci-16-00905] Morgan J. (2017). The employee experience advantage: How to win the war for talent by giving employees the workspaces they want, the tools they need, and a culture they can celebrate.

[B38-behavsci-16-00905] Murtola A. M., Vallelly N. (2023). Who cares for wellbeing? Corporate wellness, social reproduction and the essential worker. Organization.

[B39-behavsci-16-00905] Onur N., Demirel H. (2024). Investigation of work-family, family-work conflict, job satisfaction and life satisfaction of kitchen staff according to some socio-demographic characteristics. Journal of Tourism & Gastronomy Studies.

[B40-behavsci-16-00905] Orb A., Eisenhauer L., Wynaden D. (2001). Ethics in qualitative research. Journal of Nursing Scholarship.

[B41-behavsci-16-00905] Panneerselvam S., Balaraman K. (2022). Employee experience: The new employee value proposition. Strategic HR Review.

[B42-behavsci-16-00905] Patton M. Q. (2015). Qualitative research & evaluation methods.

[B43-behavsci-16-00905] Perlow L. A. (1999). The time famine: Toward a sociology of work time. Administrative Science Quarterly.

[B44-behavsci-16-00905] Pickles M., Duffy P. O. R., Stewart J. (2025). Addressing global labour challenges: An integrative model for sustainable hospitality workplaces, informed by resource-based view theory and the kaleidoscope career model. International Journal of Hospitality Management.

[B45-behavsci-16-00905] Plaskoff J. (2017). Employee experience: The new human resource management approach. Strategic HR Review.

[B46-behavsci-16-00905] Rodríguez-Modroño P., Agenjo-Calderón A., López-Igual P. (2024). A social reproduction analysis of digital care platform work. New Political Economy.

[B47-behavsci-16-00905] Saito H., Brozović D., Baum T. (2025). Well-being of hospitality employees: A systematic literature review. International Journal of Hospitality Management.

[B48-behavsci-16-00905] Saunders B., Sim J., Kingstone T., Baker S., Waterfield J., Bartlam B., Burroughs H., Jinks C. (2018). Saturation in qualitative research: Exploring its conceptualization and operationalization. Quality & Quantity.

[B49-behavsci-16-00905] Schoellbauer J., Sonnentag S., Prem R. (2022). I’d rather know what to expect: Work unpredictability as a threat to occupational well-being. Work & Stress.

[B50-behavsci-16-00905] Scholz R. W., Tietje O. (2002). Embedded case study methods: Integrating quantitative and qualitative knowledge.

[B51-behavsci-16-00905] Seyitoğlu F., Atsız O., Acar A. (2023). The future of restaurant labour: Evidence from the U.S. restaurants. Current Issues in Tourism.

[B52-behavsci-16-00905] Sinha A., Sedai A. K., Rahut D. B., Sonobe T. (2024). Well-being costs of unpaid care: Gendered evidence from a contextualized time-use survey in India. World Development.

[B53-behavsci-16-00905] Stake R. E. (1995). The art of case study research.

[B54-behavsci-16-00905] Terry G., Hayfield N., Clarke V., Braun V., Willig C., Rogers W. S. (2017). Thematic analysis. The SAGE handbook of qualitative research in psychology.

[B55-behavsci-16-00905] Timmermans S., Tavory I. (2012). Theory construction in qualitative research: From grounded theory to abductive analysis. Sociological Theory.

[B56-behavsci-16-00905] Tokumitsu M. (2015). Do what you love: And other lies about success and happiness.

[B57-behavsci-16-00905] Umney C. (2025). What does it mean to be passionate about your job? Agency, vocational habitus and self-work. Work, Employment and Society.

[B58-behavsci-16-00905] Vallerand R. J., Blanchard C., Mageau G. A., Koestner R., Ratelle C. F., Léonard M., Gagné M., Marsolais J. (2003). Les passions de l’âme: On obsessive and harmonious passion. Journal of Personality and Social Psychology.

[B59-behavsci-16-00905] Venn D., Strazdins L. (2017). Your money or your time? How both types of scarcity matter to physical activity and healthy eating. Social Science & Medicine.

[B60-behavsci-16-00905] Vieten L., Wöhrmann A. M., Wendsche J., Michel A. (2023). Employees’ work breaks and their physical and mental health: Results from a representative German survey. Applied Ergonomics.

[B61-behavsci-16-00905] Vu O. T. K., Alonso A. D., Bressan A., Tran L. N., Tran T. D., Nicholson G. J. (2023). Is the cooking profession still ‘hot’? A qualitative cross-national study of chefs’ career perceptions. Journal of Hospitality and Tourism Management.

[B62-behavsci-16-00905] Warhurst C., Nickson D. (2007). Employee experience of aesthetic labour in retail and hospitality. Work, Employment and Society.

[B63-behavsci-16-00905] Wolkowitz C. (2006). Bodies at work.

[B64-behavsci-16-00905] Ye Y., Wu L. Z., Lyu Y., Liu X. (2024). How to make the work–Family balance a reality among frontline hotel employees? The effect of family supportive supervisor behaviors. International Journal of Hospitality Management.

[B65-behavsci-16-00905] Yin R. K. (2018). Case study research and applications: Design and methods.

[B66-behavsci-16-00905] Zampoukos K. (2021). The hospitable body at work—A research agenda. Gender, Work & Organization.

